# [Retracted] MicroRNA‑454‑3p inhibits cervical cancer cell invasion and migration by targeting c‑Met

**DOI:** 10.3892/etm.2024.12467

**Published:** 2024-03-04

**Authors:** Yan Guo, Min Tao, Min Jiang

Exp Ther Med 15:2301–2306, 2018; DOI: 10.3892/etm.2018.5714

Following the publication of this paper, a concerned reader drew to the attention of the Editorial Office that, concerning the wound healing assay data shown in Fig. 4A on p. 2304, as many as five of the six data panels appeared to be overlapping, such that data which had been included in the figure to represent different experimental conditions were likely to have been derived from a much smaller number of original sources. After having conducted an independent enquiry in the Office, we were able to confirm the validity of the reader’s concern. Therefore, owing to the number of overlapping data panels that were identified and also on the basis of an overall lack of confidence in the presented data, the Editor of *Experimental and Therapeutic Medicine* has decided that this paper should be retracted from the Journal. The authors were asked for an explanation to account for these concerns, but the Editorial Office did not receive a reply. The Editor apologizes to the readership for any inconvenience caused.

